# Asymmetrical disassortative pollination mediated by long‐/short‐tongued pollinators in a distylous *Limonium myrianthum* (Plumbaginaceae) with a short corolla tubular small flower

**DOI:** 10.1002/ece3.11284

**Published:** 2024-04-21

**Authors:** Fangfang Jiao, Xiaowei Wang, Aiqin Zhang

**Affiliations:** ^1^ Xinjiang Key Laboratory of Biological Resources and Genetic Engineering, School of Life Science and Technology Xinjiang University Urumqi China

**Keywords:** floral morph variation, heterostyly, *Limonium myrianthum*, long‐ and short‐tongued pollinators, pollination patterns, small flower with short corolla tube

## Abstract

In heterostylous plants, short‐tongued pollinators are often ineffective/inefficient owing to the limitations imposed by a long corolla tube. However, it is unclear how disassortative pollen transfer is achieved in small flowers. We investigated the pollination pattern and floral morph variation by analyzing heterostylous syndrome, pollinator groups, and pollen deposition after a single visitation in two *Limonium myrianthum* populations with short‐corolla‐tubular small flowers. The predominant pollinators in the Hutubi population were pollen‐seeking short‐tongued syrphids, which can only transfer pollen between high‐level sexual organs. In the Xishan population, nectar‐seeking short‐tongued insects were efficient pollinators with symmetrical disassortative pollen transfer between high‐ and low‐level sexual organs, whereas long‐tongued pollinators had a low efficiency between high‐level sexual organs due to the low contact probability with the stigma of long‐styled flowers (L‐morph), which no longer offered the same advantage observed in tubular flowers. Asymmetrical disassortative pollination may cause the female fitness of short‐styled (S‐morph) individuals in the Hutubi and L‐morph individuals in the Xishan population to suffer greater selection pressure and exhibit a higher degree of floral morph variation. *Limonium myrianthum* exhibits an unusual pollination pattern in which the small flowers with short corolla tubes make it possible for short‐tongued insects to become effective pollinators. However, factors such as the position of stigma–anther within the flower, pollinator species and their preference further caused asymmetrical disassortative pollen transfer. Therefore, more factors should be considered when evaluating the effectiveness of short‐ and long‐tongued insects in pollination service.

## Graphical abstract

The pollination efficiency and their effects on floral morph variation of short‐tongued and long‐tongued pollinators were investigated in two *Limonium myrianthum* populations with short corolla tubular small flowers and considerable floral morph variation. We found that asymmetrical disassortative pollination affects floral morph variation. Compared with nectar‐seeking ST insects, pollen‐seeking ST insects and LT insects caused asymmetrical disassortative pollen transfer between high‐ and low‐level sexual organs due to the preference for reward and the position of stigmas/anthers within the flower.

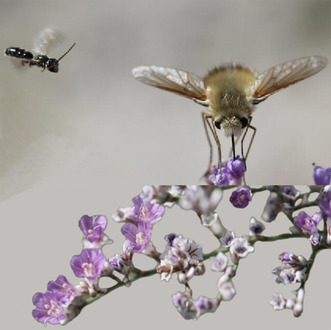

## 1 INTRODUCTION

Although debatable (Vamosi et al., 2018; Wilson & Thomson, 1996), it is widely accepted that the coevolution of angiosperms and pollinators has resulted in a great diversity of flowering plants and pollinators (Cha et al., 2023; Ruchisansakun et al., 2021; Whittall & Hodges, 2007; Zhang, 2004). Pollination is required for the successful reproduction of flowering plants. Approximately 90% of angiosperms are pollinated by animals (Ollerton et al., 2011; Stephens et al., 2023). However, researchers disagree on the frequency and intensity of pollinator selection events in nature (Aigner, 2001; Herrera, 2001).

Heterostyly is a genetically controlled floral polymorphism that includes both distyly and tristyly (Barrett, 2019; Barrett & Shore, 2008; Darwin, 1877; Webb & Lloyd, 1986). It is thought to be a plant adaptation that promotes accurate disassortative pollen transfer through reciprocal herkogamy between morphs, facilitating pollen deposition on the different parts of the pollinator's body surface (Brys & Jacquemyn, 2020; Darwin, 1877; Deschepper et al., 2018; Furtado et al., 2021; Raupp et al., 2020). Reciprocal herkogamy is usually accompanied by a sporophytically controlled heteromorphic incompatibility that prevents self‐fertilization and intramorph fertilization, as well as a suite of ancillary morphological polymorphisms, such as stigma and pollen polymorphism (Barrett, 1990; Ganders, 1979; Lloyd & Webb, 1992a). Such floral polymorphisms and pollen transfer models provide a paradigmatic system for studying plant–pollinator interactions.

Ganders (1979) reported that floral features of heterostyly were common across different families, including moderately sized flowers, reliance on long‐tongued (LT) pollinators, radial corolla and long corolla tubes with nectar concealed at the base, and low‐level sexual organs inside the tube (Ganders, 1979; Simón‐Porcar et al., 2024). The causes of such common floral traits are unknown; however, the traits may be due to developmental and functional constraints (Barrett, 1992). For example, in small flowers, compared to most pollinator sizes and their distance of movement within flowers, the degree of herkogamy (stigma–anther separation) may be negligible, making the accurate transfer of legitimate pollen difficult. This is referred to as “disordered herkogamy” (Webb & Lloyd, 1986; Zhang, 2004). Similarly, in large flowers, it is difficult to ensure that high‐ and low‐level sexual organs make contact with the body surfaces of most pollinators simultaneously due to excessive stigma–anther separation. Only moderately moderately‐sized flowers, with flower lengths typically ranging from 5 to 30 mm, may preserve stigma–anther separation and allow high and low‐level sexual organs to make contact with different parts of the pollinator's body surface simultaneously due to conducive stigma–anther separation (Barrett, 1992; Brys & Jacquemyn, 2020; Deschepper et al., 2018; Furtado et al., 2021; Oliveira et al., 2022; Raupp et al., 2020).

In terms of flower shape, a long corolla tube may be associated with accurate pollen transfer through limiting visiting behavior and stigma–anther position within a flower. Due to the longer corolla tube, LT insects are frequently ideal pollinators for the maintenance of isoplethic floral morph frequency in populations, whereas short‐tongued (ST) pollinators are usually ineffective or inefficient. Because ST pollinators only come into contact with high‐level sexual organs when collecting pollen, long‐styled morph (L‐morph) flowers usually have a relatively high reproductive fitness or floral morph frequency in a population dominated by ST pollinators (Arroyo et al., 2002; Arroyo & Dafni, 1995). For example, the loss of short‐styled morph (S‐morph) flowers in *Eichhornia azurea* (Pontederiaceae) may be related to a lack of LT pollinators in coastal populations (Alves dos Santos, 2002). Because L‐morph flower pollen cannot be transferred to S‐morph flower stigmas by ST pollinators, the population with a higher visiting frequency of ST pollinators has more L‐morph plants in *Narcissus papyraceus* (Amaryllidaceae) (Pérez‐Barrales & Arroyo, 2010; Simón‐Porcar et al., 2014). Such studies have demonstrated that corolla tube length has a restrictive influence on ST insect visiting behavior and symmetrical pollen transfer between floral morphs.

Although moderately‐sized tubular flowers are more common in heterostylous plants, there are many small flowers without corolla tubes or with short corolla tubes whose stigmas and anthers are located above the short corolla tube, such as bowl‐shaped flowers, dish‐shaped flowers or semi‐tubular flowers, in some Polygonaceae and Plumbaginaceae species (Barrett, 2019; Wu et al., 2017). Such species, which typically have very small flowers, lack the limitations of the corolla tube or have low constraints on pollinator visiting behavior. The roles that LT/ST pollinator functional groups play in maintaining floral morph frequency and reproductive fitness in populations with small flowers are worth studying.

*Limonium myrianthum* (Plumbaginaceae), a perennial herb with small flowers and stigmas and anthers located above the short corolla tube, is found in salty desert environments. According to the results of preliminary investigations, *L. myrianthum* is a distylous plant with dimorphism in pollen–stigma morphology and considerable floral morph variation. In addition, some populations only have ST pollinators, while others have both LT and ST pollinators. Floral morph variation has been hypothesized to be linked to different pollinator functional groups or/and floral syndromes, which has received a lot of attention in medium‐sized tubular flowers (Pérez‐Barrales & Arroyo, 2010; Simón‐Porcar et al., 2014; Yuan et al., 2017). However, in small flowers with short corolla tubes, it is not clear what pollen transfer patterns are caused by LT and ST pollinators and how they influence floral morph variation. To that end, the aim of the present study was to address two main questions in two populations with different pollinator functional groups: (1) What are the heterostylous syndromes of *L. myrianthum* and the degree of variation of L‐ and S‐morph flowers? To answer the question, aspects of the heterostylous syndrome, including floral size, ancillary polymorphism, and heteromorphic incompatibility, were examined. Additionally, floral morph variation was investigated by measuring stigma–anther height and calculating the coefficient of variation of L‐ and S‐morph flowers in two populations; (2) how is disassortative pollen transfer achieved mediated by LT and ST pollinators? To answer the question, visiting behavior and pollination efficiency were compared by quantifying legitimate pollen on stigmas after a single visit by LT and ST pollinators in two populations. Based on the studies, the roles of LT and ST pollinators in promoting disassortative pollination and the relationship between flower morphological features and pollinator functional groups are explored.

## 2 MATERIAL AND METHODS

### 2.1 Study site and species

*Limonium myrianthum* has a large paniculate inflorescence with one to several inflorescence axes and 3–5 round branches and grows to a height of 40–100 cm. The flower has a radially symmetrical blue‐purple corolla and a narrow funnelform calyx, with five anthers and filiform stigmas protruding over a short corolla tube. It has one locule and one ovule in the ovary, and the fruit is wrapped in a persistent calyx. The flowering period is from June to July.

**FIGURE 1:**
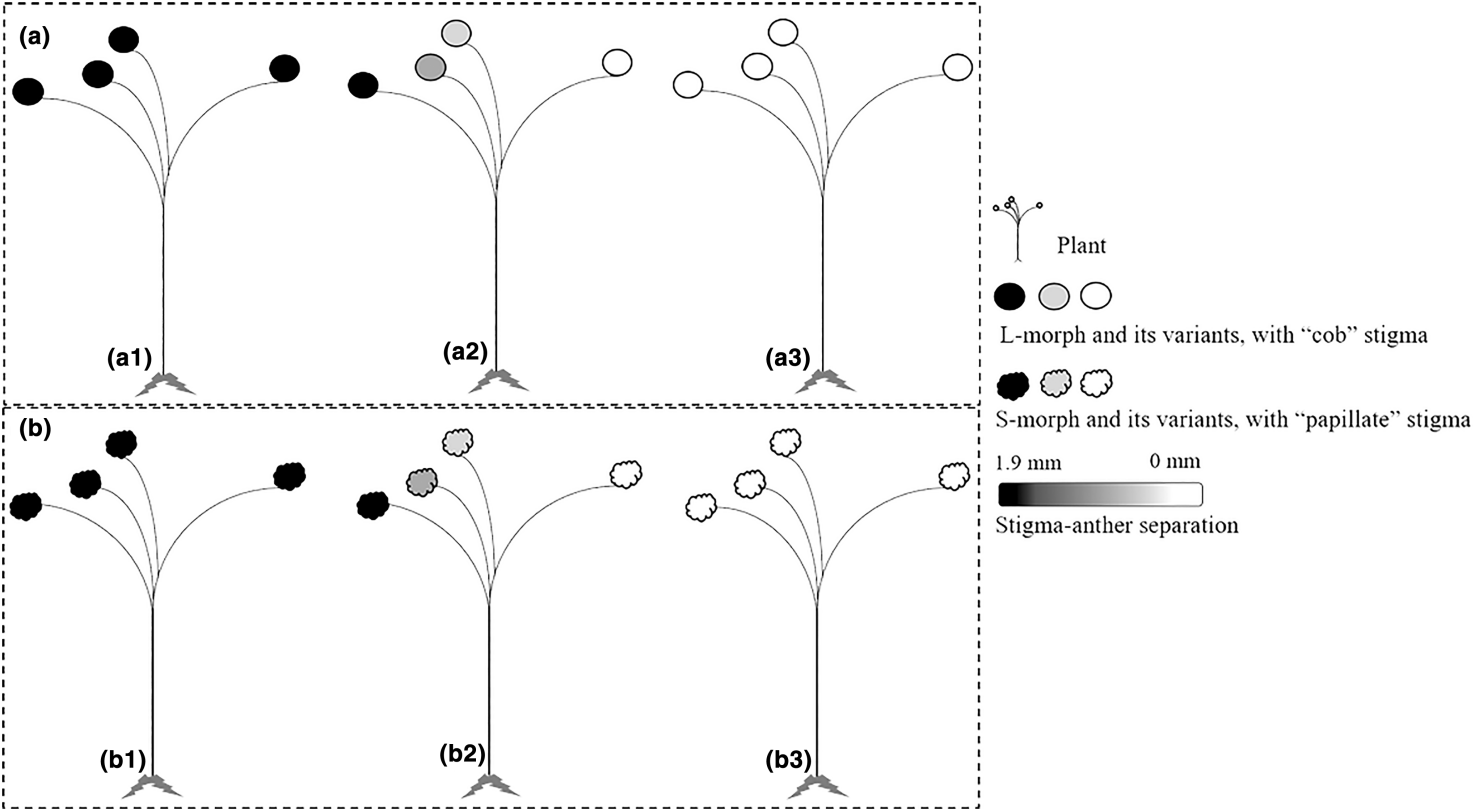
Two types of plants and floral morphs with different pollen–stigma morphologies in two populations. (a1–a3) The plants and floral morphs with “cob” stigma morphology (icon: ○); (b1–b3) the plants and floral morphs with “papillate” stigma morphology (icon: 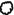). The deeper the icon color, the larger their stigma–anther separation.

An ST pollinator‐dominated population at Hutubi (HTB) with plant heights of 60–100 cm and an LT and ST pollinator‐dominated population at Xishan (XS) with plant heights of 40–60 cm were selected in Xinjiang, northwestern China. Based on our field observations of two populations, there were great floral morph variations in some L‐ and S‐morph plants, in which stigma–anther separation of two floral morphs tends to decrease, and homostyly with identical or nearly identical stigma and anther height were common, forming the populations consisting of L‐morph, S‐morph flowers, and their variants (homostyly) (Figure 1). In addition, there were two types of pollen–stigma morphology in each population, with L‐morph flowers and their variants of one type (Figure 1a1–a3) and S‐morph flowers and their variants being another type (Figure 1b2,b3), with a 1:1 morph ratio.

### 2.2 Floral traits, floral morph variation, and ancillary polymorphism

To investigate the floral traits of L‐ and S‐morph flowers of *L. myrianthum*, 20 flowers of each morph were harvested randomly from different individuals. Digital calipers were used to measure corolla open diameter, corolla tube length, stigma, and anther height (accuracy of 0.02 mm).

To investigate the degree of variation in floral morphs, 50 L‐ and S‐morph flowers and their variants were selected randomly from 50 different individuals from two populations. The stigma and anther height were measured using digital calipers, and the coefficient of variation was calculated based on the stigma–anther separation.

To examine pollen and stigma morphology, 10 individuals were selected randomly per floral morph in two populations, collecting 1–2 nearly open floral buds from each individual, and then the following processes were performed: anthers were air‐dried naturally in EP tubes, while stigmas were fixed in 2% glutaraldehyde fixative (0.1 mol L^−1^ phosphate buffer). A scanning electron microscope (LEO 1430 VP; Carl Zeiss, Oberkochen, Germany) was used for observation and photography.

### 2.3 Pollinator groups and visiting frequency

To examine the pollination environments in different populations, pollinator species and visiting behavior were observed between 10:00 am and 5:00 pm on sunny, windless days, for not <0.5 h per observation. Four to five plants with different floral morphs were selected randomly, and two to three branches were marked. The number of flowers per branch and the number of flowers visited were recorded. The visiting frequency was obtained as the total number of flowers visited divided by the number of open flowers observed and the total number of hours per observation period.

### 2.4 Stigmatic pollen deposition after a single visit by LT and ST pollinators

**FIGURE 2:**
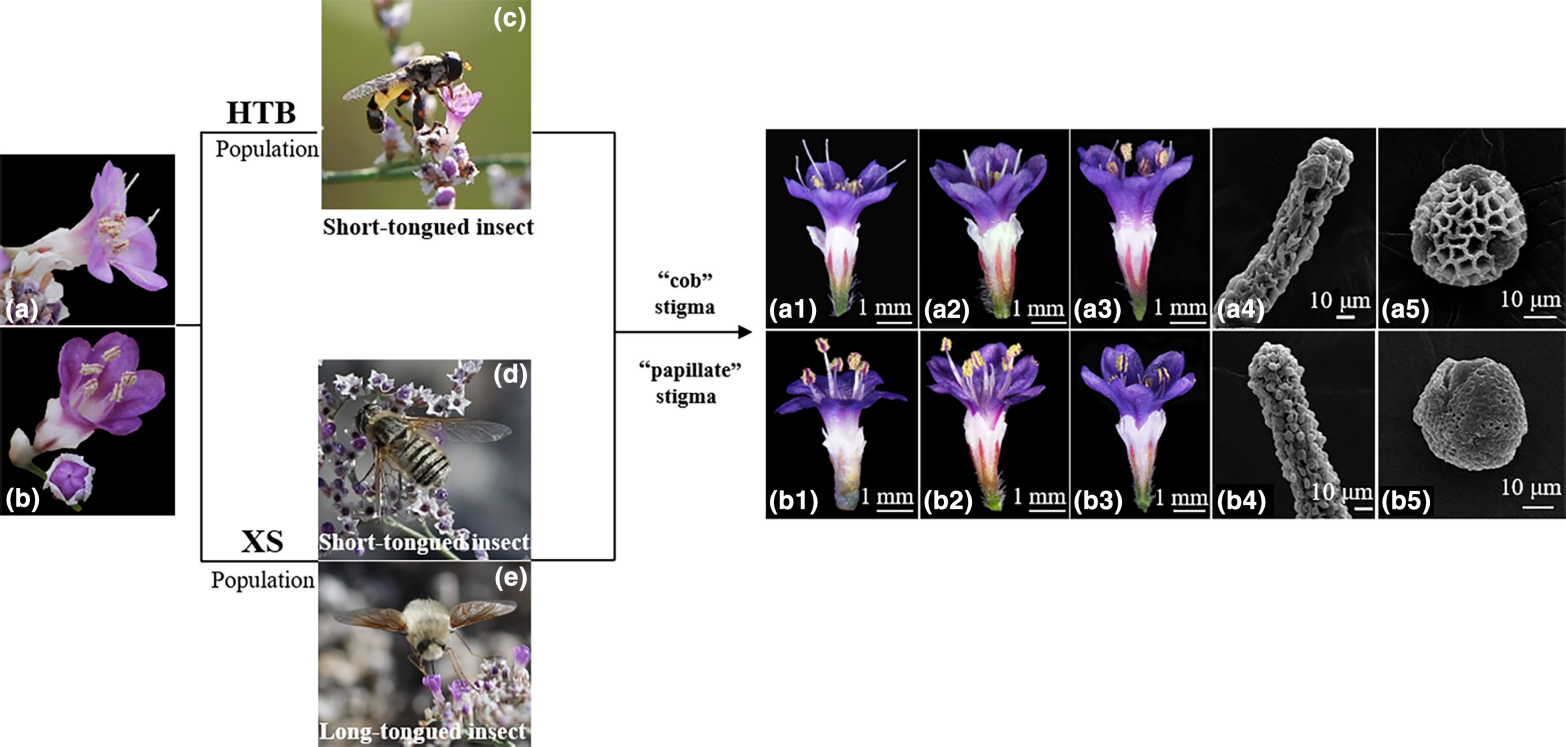
Floral morphs, morphology of stigmas and pollen grains, and pollinators of *Limonium myrianthum*. (a) Long‐styled morph flower; (b) short‐styled morph flower; (c) the short‐tongued pollinator Syrphidae in Hutubi population; (d) the short‐tongued pollinator Muscidae in Xishan population; (e) the long‐tongued pollinator *Bombylius* sp. in Xishan population; (a1–a5) the long‐styled morph flowers and their variants, and their “cob” stigma and coarse reticulate pollen morphology; (b1–b5) the short‐styled morph flowers and their variants, and their “papillate” stigma and finely reticulate pollen morphology.

To test the pollen transfer efficiency of LT and ST pollinators, 15 individuals with different floral morphs were labeled randomly in two populations during the flowering peak. Two to three twigs with buds about to open were selected randomly for bagging to prevent them from pollinators in each individual. After most flowers had opened, the bags were removed to await pollinator visits. Visited flowers were documented and removed using tweezers, and the floral morphs and visitor species were recorded. Pollen deposition on the stigmas of L‐morph and S‐morph flowers was counted. The legitimate pollen were identified by a particular exine sculpture (Figure 2a5,b5). Data were collected for 3 days in the XS population and 5 days in the HTB.

### 2.5 Heteromorphic incompatibility system

To assess heteromorphic incompatibility of *L. myrianthum*, 15 L‐morph and S‐morph plants were labeled randomly, respectively, at the HTB population flowering peak, and 15 flowers were selected randomly from each plant for the following treatments (2–3 flowers per treatment): (1) emasculation (to test apomixis); (2) hand intramorph pollination (L × L, S × S); (3) hand intermorph pollination (S × L, L × S); (4) hand self‐pollination; and (5) control (flowers were pollinated naturally). All treatments were bagged, except for the control. The fruit sets of different pollination treatments were evaluated after the fruit ripened.

### 2.6 Statistical analyses

Floral traits of floral morphs were compared within and between populations using a generalized linear model (GLM) with a Gaussian distribution using the “log” link function to improve normality using the “glm” function in the “lme4” package in R (Bates et al., 2015). Population, morph, and their interactions were used as predictive variables, and floral traits as the response variable. The significance of each factor was tested with a type II analysis of deviance, which was conducted using the “Anova” function in the “car” package (Fox & Weisberg, 2019). The “emmeans” function with a Tukey adjustment from the package “emmeans” (Lenth, 2023) was used as a post‐hoc test to detect significant differences between morphs. To examine the differences in sexual organ height within/between floral morphs of two populations, population, morph, sex organ type, and morph × sex organ type were used as predictive variables, with height of sex organs as a response variable.

The visiting frequencies of pollinators were analyzed using linear mixed‐effects models (LMMs) to investigate the effects of pollinators on visiting frequencies. Before statistical tests, visiting frequency was transformed with log_10_ (*x* + 1) to improve data normality (Zar, 2010). Different days of observation were included as a random factor. The analyses were conducted using the “lmer” function in the “lme4” package (Bates et al., 2015). To obtain the significance of pollinator groups, a type II analysis of variance was conducted using the “Anova” function in the “car” package (Fox & Weisberg, 2019). The “emmeans” function with a Tukey adjustment from the package “emmeans” (Lenth, 2023) was used as a post‐hoc test to detect significant differences between pollinator groups.

The legitimate pollen was analyzed using generalized linear mixed‐effects models (GLMMs) to investigate the effects of morph, pollinator, and interaction term on legitimate pollen deposition on stigma after a single visit. The number of legitimate pollen was modeled with a Poisson distribution using the “log” link function. The analyses were conducted using the “glmer” function in the “lme4” package in R (Bates et al., 2015). Morph, pollinator, and interaction terms were included in the models as fixed factors, and individual plants nested within different days for observation were defined as a random factor. Due to the fact that the HTB population only has short‐tongued pollinators, only the morph is used as a fixed effect in the model. The significance of each factor was tested with a type II analysis of variance (ANOVA) as described above.

Heteromorphic incompatibility was analyzed using generalized linear mixed‐effects models (GLMMs) to investigate the effects of treatment, morph, and interaction terms on fruit set. Individual plants were included as a random factor. The fruit set was modeled with a binomial distribution using the “logit” function as described above. To obtain the significance of each factor, a type II ANOVA was conducted.

All statistical analyses in the present study were performed in R software version 4.2.3 (R Core Team, 2023). All figures are created using GraphPad Prism 9.5.1 (GraphPad Software Inc., San Diego, CA, USA). All data are presented as mean ± SE.

## 3 RESULTS

### 3.1 Floral traits, floral morph variation, and ancillary polymorphism

Based on the measurements of flower sizes of the L‐ and S‐morph flowers, there were no significant differences in corolla open diameter (HTB: *p* = .578; XS: *p* = .911) or corolla tube length (HTB: *p* = .950) between the L‐ and S‐morph flowers within a population (Tables 1 and 2), except for the XS population, whose corolla tubes in the L‐morph were larger than those in the S‐morph (*p* = .044). However, the stigma and anther heights were significantly different between L‐morph (HTB: *p* < .001; XS: *p* < .001) and S‐morph flowers (HTB: *p* < .001; XS: *p* < .001). No significant differences were observed between the high‐level sexual organs of L‐ and S‐morph flowers (HTB: *p* = .999; XS: *p* = .999), but significant differences were noted between the low‐level sexual organs, in which the anthers of L‐morph flowers were significantly higher than the stigmas of S‐morph flowers (HTB: *p* < .0001; XS: *p* < .0001) (Tables 1 and 3).

**TABLE 1:** Floral size of *Limonium myrianthum* in two populations.

Floral trait (mm)	Floral morph	HTB	XS
		Mean ± SE	Mean ± SE
Corolla open diameter	L	2.60 ± 0.03^aA^	2.47 ± 0.06^aA^
	S	2.64 ± 0.03^aA^	2.48 ± 0.06^aB^
Corolla tube length	L	3.32 ± 0.11^aA^	2.38 ± 0.06^aB^
	S	3.31 ± 0.05^aA^	2.13 ± 0.04^bB^
Stigma height	L	5.19 ± 0.05^aA^	4.76 ± 0.08^aB^
	S	3.82 ± 0.07^bA^	3.59 ± 0.08^bB^
Anther height	L	4.21 ± 0.07^aA^	4.03 ± 0.06^aA^
	S	5.17 ± 0.05^bA^	4.77 ± 0.11^bB^

*Note*: In the same row, different capital letters indicate the significant difference of the same floral morph between populations; in the same column, different lowercase letters indicate the significant difference between floral morphs within the populations.

**TABLE 2:** Comparison of floral size within/between populations using generalized linear model (GLM).

Dependent variable	Source	df	*χ* ^2^	*p*
Corolla open diameter	Population	1	9.207	**.002**
	Morph	1	0.235	.628
	Population × morph	1	0.089	.765
Corolla tube length	Population	1	152.727	**<.001**
	Morph	1	1.438	.230
	Population × morph	1	2.790	.095
Stigma height	Population	1	23.21	**<.001**
	Morph	1	327.43	**<.001**
	Population × morph	1	0.53	.466
Anther height	Population	1	16.375	**<.001**
	Morph	1	132.60	**<.001**
	Population × morph	1	1.191	.275

*Note*: The significance of bold values is that the variation between different treatments has significant difference.

**TABLE 3:** Comparison of height of stigma and/or anther between floral morphs in different populations using generalized linear model (GLM).

Dependent variable	Source	df	*χ* ^2^	*p*
Sex organ height	Population	1	39.22	**<.001**
	Morph	1	13.36	**<.001**
	Sex organ type	1	10.66	**.001**
	Morph × sex organ type	1	438.60	**<.001**

*Note*: The significance of bold values is that the variation between different treatments has significant difference.

Between populations, the floral size of every morph, including the corolla open diameter, corolla tube length, height of stigma, and height of anther in the HTB population, were greater than those in the XS population (Tables 1 and 2). The corolla open diameter and corolla tube length were significantly affected by populations but were not significantly affected by morph, nor was the interaction between morph and population (Tables 1 and 2). The stigma and anther heights were affected significantly by morph and population, but their interaction was not significant (Table 3).

The degrees of floral morph variation in the L‐ and S‐morph flowers were significantly different in the two populations. In the HTB population, the degree of variation was higher in the S‐morph flowers, with a coefficient of variation of 0.79, compared to L‐morph flowers (0.40). In the XS population, L‐morph flowers exhibited a greater degree of variation, with a coefficient of variation of 0.74, compared to S‐morph flowers (0.65).

The pollen ornamentation and stigma mastoid cells were all dimorphic in the two populations, with the L‐morph and its variants all having cob‐like stigma epidermal cells (hereafter “cob” stigma) and coarse reticulate pollen exine ornamentation (Figure 2a4,a5), and the S‐morph and its variants all having papillate stigma epidermal cells (hereafter “papillate” stigma) and finely reticulate pollen exine ornamentation (Figure 2b4,b5).

### 3.2 Pollinator groups and visiting frequency

**FIGURE 3:**
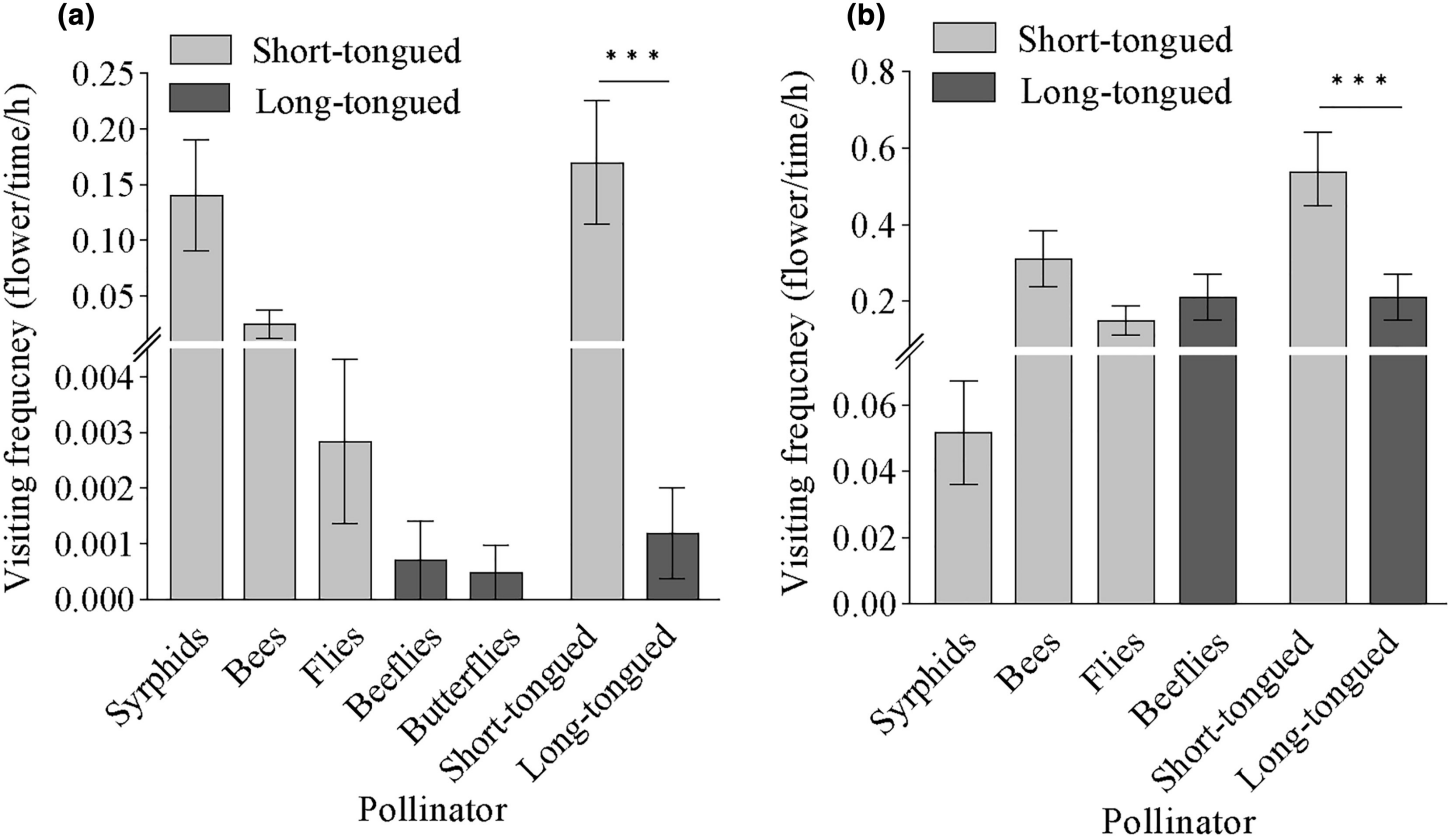
Visiting frequency of pollinators of *Limonium myrianthum* in two populations (bars indicate mean ± SE). (a) Hutubi; (b) Xishan. Bars with asterisks indicate a significant difference between pollinator groups (***p < .001) according to linear mixed‐effects models (LMMs).

The two populations had different pollinator functional groups based on the observation and analysis of pollinator species and visiting behavior. Our 17.25‐h observation was recorded for two consecutive days in the HTB population. ST pollinators were the most active pollinator group in the HTB population, with syrphids being the primary pollinators with a visiting frequency of 0.14 ± 0.05 flower time^−1^ h^−1^ (Figure 2c), followed by small bees (0.025 ± 0.001 flower time^−1^ h^−1^) and flies (0.003 ± 0.001 flower time^−1^ h^−1^). The LT pollinators *Bombylius* spp. and butterflies appeared only once during the observation period (Figure 3a).

Our 31.91‐h of observation was recorded for five consecutive days in the XS population. There were two types of pollinators in the XS population: LT and ST pollinators included bees, flies, and syrphids with a total visiting frequency of 0.54 ± 0.10 flower time^−1^ h^−1^ (Figure 2d); *Bombylius* spp. was the main LT pollinator with a visiting frequency of 0.21 ± 0.06 flower time^−1^ h^−1^ (Figure 2e). The visiting frequency of ST pollinators was significantly higher than that of LT pollinators (Wald*χ* ^2^ = 10.122, *p* < .01) (Figure 3b).

### 3.3 Legitimate pollen deposition on stigmas by a single visit of LT/ST pollinators

**FIGURE 4:**
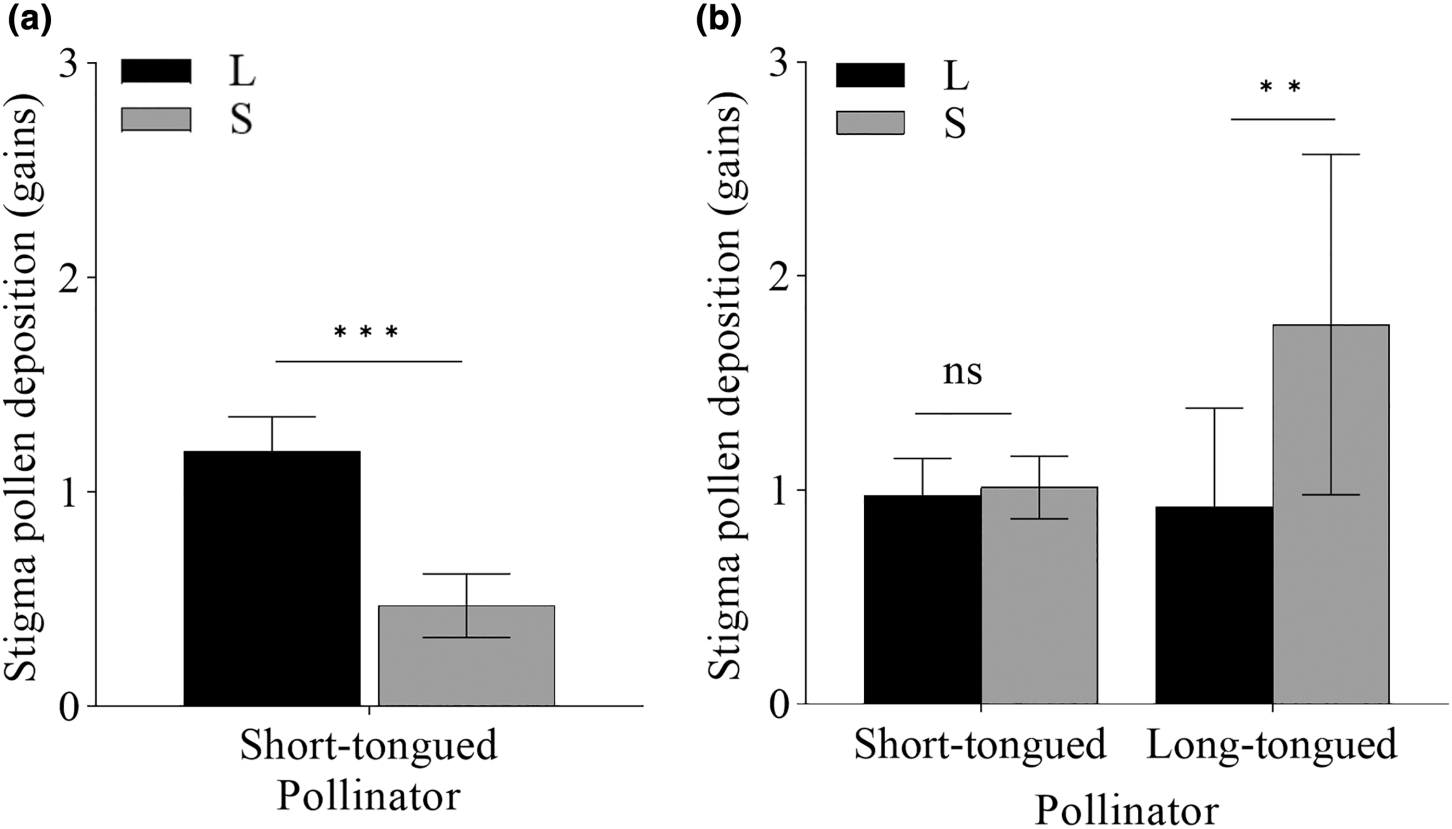
Number of legitimate pollen grains on stigmas after a single visitation of long‐tongued/short‐tongued pollinators in two populations of *Limonium myrianthum* (bars indicate mean ± SE). (a) Hutubi; (b) Xishan. Bars with asterisks indicate a significant difference between morphs (***p < .001; **p < .01; ns, *p* > .05) according to generalized linear mixed‐effects models (GLMMs).

Based on stigmatic pollen deposition after a single visit statistics, the legitimate pollen grains on the stigmas of L‐ and S‐morph flowers were 1.19 ± 0.16 and 0.47 ± 0.15, respectively, when they were visited by ST pollinators in the HTB population. The difference between floral morphs was significant (Wald*χ* ^2^ = 17.721, *p* < .001). However, LT pollinators were rare, and no data were collected during the observation period (Figure 4a).

In the XS population, legitimate pollen deposition was significantly affected by morph (Wald*χ* ^2^ = 5.333, *p* = .021) and pollinator (Wald*χ* ^2^ = 10.093, *p* = .001), but was not affected significantly by their interaction on pollen deposition (Wald*χ* ^2^ = 1.917, *p* = .166). ST pollinators brought a total of 0.97 ± 0.18 and 1.01 ± 0.15 legitimate pollen grains to the stigmas of L‐ and S‐morph flowers, respectively, with no significant difference between morphs (*p* = .142). The primary LT pollinator, *Bombylius* spp., delivered 0.92 ± 0.46 and 1.77 ± 0.80 legitimate pollen grains to the stigmas of L‐ and S‐morph flowers, respectively. The pollen grains in the stigmas of S‐morph flowers were significantly more numerous than those in L‐morph flowers (*p* = .0056) (Figure 4b).

### 3.4 Heteromorphic incompatibility system

**FIGURE 5:**
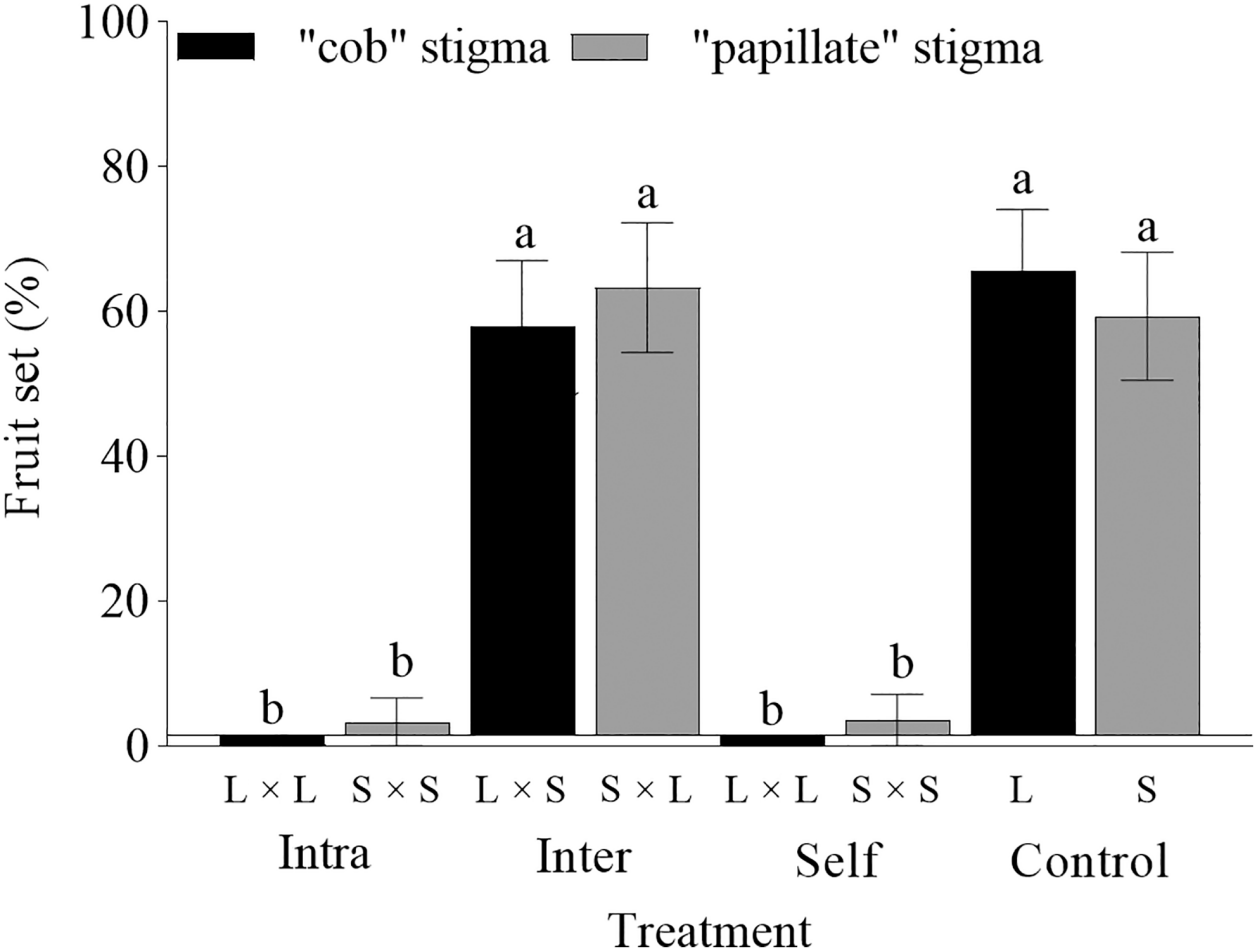
The fruit set under different pollination treatments (bars indicate mean ± SE). Same letters above the bars indicate that there is no statistically significant difference. Different letters indicate a statistically significant difference (*p* < .01) according to generalized linear mixed‐effects models (GLMMs).

The *L. myrianthum* species exhibited no apomixis, self, or intramorphic compatibility, so the flowers produced no or a few fruits, after emasculation and bagging, as well as intramorphic and artificial self‐pollination. Under intermorphic‐pollination, such as L × S (58.06 ± 9.01%) and S × L (63.33 ± 8.95%), there was no difference between floral morphs in the fruit set (*p* = .687) (Figure 5).

Under open pollination, there was no difference between floral morphs in the fruit set (L‐morph: 65.63 ± 8.53%, S‐morph: 59.38 ± 8.82%, *p* = .578), which was consistent with that of intermorph pollination. The floral morph (Wald*χ* ^2^ = 0.645, *p* = .724) and floral morph × treatment (Wald*χ* ^2^ = 0.635, *p* = .959) had no significant effect on fruit set, whereas treatment (Wald*χ* ^2^ = 24.068, *p* < .001) had a significant impact.

## 4 DISCUSSION

Heterostyly is frequently associated with LT pollinators and moderately‐sized tubular flowers (Ganders, 1979; Lloyd & Webb, 1992b; Simón‐Porcar et al., 2024). Owing to the limitation of long corolla tubes, ST pollinators often become ineffective or inefficient pollinators and drive floral morph frequency deviation, or loss of floral morphs (Simón‐Porcar et al., 2014). In *L. myrianthum*, an unusual pollen transfer pattern is revealed due to the effects of the small flower with a short corolla tube, visiting preference, and position of the stigma and anther. It indicates that the main pollinators, pollen‐seeking ST insects (syrphids), promoted disassortative pollination between high‐level sexual organs and asymmetrical pollen flow between high and low sexual organs in the HTB population. However, in the XS population, nectar‐seeking ST insects caused symmetrical disassortative pollen transfer between high‐ and low‐level sexual organs. And asymmetrical pollen flow was caused by LT insects due to low contact efficiency with stretched stigmas in L‐morph flowers.

The reproductive fitness of plants is closely associated with floral traits and pollinator behaviors (Dafni & Kevan, 1997; Wu et al., 2018; Zhu et al., 2015). In heterostyly, when visiting a flower without restraining the corolla tube, pollinators can approach nectaries from any direction. Therefore, pollinator behavior may be critical to achieving accurate pollen transfer between floral morphs (Lloyd & Webb, 1992b). Our study results confirmed this inference. Judging from the pollination patterns of LT and ST insects, small flowers with short corolla tubes make it possible for ST insects to become effective pollinators. However, different visiting behaviors (feeding preferences) of ST insects caused completely different pollen flow between floral morphs in the two populations. For example, in the HTB population, the main pollinators, pollen‐seeking syrphids, brought more legitimate pollen to L‐morph flowers than to S‐morph flowers. However, in the XS population, ST pollinators, including syrphids, bees, and flies, brought the same number of legitimate pollen to L‐ and S‐morph flowers after a single visit due to most of them feeding on nectar. The results suggest that the different pollination patterns and efficiency of ST insects were related to many factors, including pollinator species, visiting behavior, flower size and shape (Armbruster et al., 2006; Arroyo et al., 2002), and habitat factors (Abdusalam et al., 2022; Deschepper et al., 2018; Santos‐Gally, Pérez‐Barrales, et al., 2013), besides corolla tube length (Arroyo et al., 2002; Arroyo & Dafni, 1995). Meanwhile, the disadvantages of ST insects cannot be ignored, which often cause excessive self‐pollen deposition (Björkman, 1995) and even become pollen thieves (Simón‐Porcar et al., 2014).

LT insects are often efficient pollinators of both floral morphs as they remove little pollen from anthers but deposit comparatively large amounts on stigmas and promote symmetrical disassortative pollen transfer due to the common probability of contact with high‐ and low‐level sex organs (Simón‐Porcar et al., 2014). However, in the XS population, a different pollen transfer pattern was presented, in which asymmetrical pollen flow was caused by LT insects due to low contact efficiency with stretched stigmas. The phenomenon differed from the results reported for heterostylous taxa with tubular flowers (Deschepper et al., 2018; Pérez‐Barrales & Arroyo, 2010; Santos‐Gally, Pérez‐Barrales, et al., 2013; Simón‐Porcar et al., 2014; Yuan et al., 2017). In most tubular flowers, the low‐level sexual organs are usually located in the corolla tube, and the high‐level sexual organs are located in the throat of the corolla tube (Zhang et al., 2014), which makes stigma and anther located in the channel of visiting flowers and come into contact with pollinators easily. In *L. myrianthum*, the stigma and anther were located above the short corolla tube (Figure 2a,b). The high‐level sexual organs had a more dispersed spatial distribution than the low‐level sexual organs, which reduced the contact opportunity with LT pollinators, resulting in asymmetry in pollen flow. Based on the coefficient of variation of the two floral morphs, the variation in L‐morph flowers was greater than that of S‐morph flowers.

Effective pollen transfer between plants depends on the pollinator species and plant regulation of the pollinator's visiting behavior (Wu et al., 2018; Zhang, 2004). For example, in *Primula secundiflora*, nectar‐robbing pollinators tend to pollinate only S‐morph flowers, whereas syrphids visit L‐morph flowers frequently (Zhu et al., 2015). In *N. papyraceus*, narrow and long corolla tubes drove heterostyly evolution (Santos‐Gally, Gonzalez‐Voyer, & Arroyo, 2013). Due to flower shape and the differences in the spatiotemporal distribution of pollinator groups, the pollination pattern in flowering plants exhibits high diversity and uncertainty (Johnson, 1996; Oliveira et al., 2022; Waser et al., 1996; Wenzell et al., 2023; Wu et al., 2018). Our study showed that the small flower with a short corolla tube could reduce the restriction on ST pollinators and make them more effective pollinators in *L. myrianthum*. However, the degree of restriction is highly variable and uncertain owing to the influence of pollinator species and visiting behavior. Therefore, more factors should be considered when evaluating the effectiveness of ST and LT in pollination services of insects based on our research and related literature.

## 5 CONCLUSION

Plant‐pollinator interactions promote and maintain species diversity. Heterostyly provides a paradigmatic system for investigating the relationship between plants and pollinators (Barrett, 2019). As a distylous species with small flowers, *L. myrianthum* exhibits an asymmetrical disassortative pollen transfer model mediated by LT/ST pollinators, which is associated with foraging behavior under ST insect visits and the spatial distribution of female–male within flowers under LT insect visits. The phenomenon is distinct from the traditional pollination patterns in tubular flowers, in which ST insects are no longer ineffective pollinators due to small flowers with short corolla tubes. Based on the different coefficients of variation between floral morphs, asymmetric pollen flow may cause greater selection pressure on the female–male fitness of the L‐ and S‐morph flowers. The pollination pattern, floral morph variation, the visiting behavior of LT and ST pollinators, and related influencing factors were revealed for the first time in *L. myrianthum*. Considering the diversity of floral traits and differences in the spatiotemporal distribution of pollinator groups, a more extensive investigation is required to obtain a more comprehensive understanding of issues such as the occurrence of homostyly, floral morph variation, and the function of reciprocal herkogamy in taxa with small flowers.

## AUTHOR CONTRIBUTIONS

**Fangfang Jiao:** Conceptualization (equal); data curation (equal); formal analysis (equal); funding acquisition (supporting); investigation (equal); methodology (equal); project administration (lead); software (equal); visualization (equal); writing – original draft (equal). **Xiaowei Wang:** Investigation (equal); methodology (equal). **Aiqin Zhang:** Conceptualization (equal); formal analysis (equal); funding acquisition (equal); methodology (equal); project administration (lead); resources (equal); supervision (equal); validation (equal); writing – review and editing (equal).

## Supporting information


Data S1.

## Data Availability

The datasets used and/or analyzed during the current study are available in the Supporting Information.
